# Responses of microbial necromass carbon and microbial community structure to straw- and straw-derived biochar in brown earth soil of Northeast China

**DOI:** 10.3389/fmicb.2022.967746

**Published:** 2022-09-23

**Authors:** Qiang Sun, Xu Yang, Zhengrong Bao, Jian Gao, Jun Meng, Xiaori Han, Yu Lan, Zunqi Liu, Wenfu Chen

**Affiliations:** ^1^Postdoctoral Station of Agricultural Resources and Environment, Land and Environment College, Shenyang Agricultural University, Shenyang, China; ^2^Key Laboratory of Biochar and Soil Improvement, Ministry of Agriculture and Rural Affairs, Shenyang, China; ^3^National Biochar Institute, Shenyang Agricultural University, Shenyang, China; ^4^Agronomy College, Shenyang Agricultural University, Shenyang, China

**Keywords:** biochar, straw, microbial necromass carbon, phospholipid fatty acids, carbon sequestration

## Abstract

Soil microbial organisms are conducive to SOC sequestration. However, little attention has been given to the contributions of living MBC and microbial necromass carbon to the SOC pool under biochar and straw amendments. The aims of the study were to explore (1) the effects of maize straw and biochar on MBC, POC, MAOC, DOC and microbial necromass carbon; (2) the contribution of MBC and microbial necromass carbon to the SOC pool; and (3) the relationships among the soil microbial community structure, microbial necromass carbon and other SOC fractions under maize straw and biochar application for nine consecutive years. Three treatments were studied: CK (applied chemical fertilizer only), BC (biochar applied annually at a rate of 2.625 t ha^−1^ combined with chemical fertilizer), and SR (straw applied annually at a rate of 7.5 t ha^−1^). Both biochar and straw increased the SOC contents after nine successive maize plant seasons; the DOC and MAOC contents were also increased by biochar and straw amendments. Biochar had advantages in increasing POC contents compared to straw. Biochar and straw increased MBC contents by 48.54% and 60.83% compared to CK, respectively. Straw significantly increased the Galn, GluN, MurA, ManN and total amino contents (*P* < 0.05); however, biochar significantly increased the Galn and GluN contents (*P* < 0.05) but had no impact on the MurA contents and decreased the ManN contents. Biochar mainly increased the fungal-derived necromass carbon contents but had no effect on the bacterial-derived necromass carbon, and straw increased both the bacterial- and fungal-derived necromass carbon contents. Straw had no influence on the ratios of microbial necromass carbon accounting for SOC and MAOC, but biochar decreased the ratios in the current study. Similarly, biochar mainly increased the fungal PLFA and total PLFA contents compared to CK, but straw increased bacterial PLFAs, fungal PLFAs and Actinomycetes PLFAs. Maize yield were increased by 7.44 and 9.16% by biochar and straw application, respectively. These results indicate that biochar stimulates fungal activities and turnover to contribute to the stable soil carbon pool and that biochar also improves POC contents to improve the soil organic carbon sink.

## Introduction

The soil organic carbon (SOC) pool is the largest terrestrial carbon pool compared to the atmospheric and vegetation carbon pools (Stockmann et al., 2013). SOC concentration and storage are vital to soil fertility, climate change mitigation and food security (Lal, 2006, 2016). SOC can be separated from stable and active carbon pools by its nature. However, the mechanisms of the stable carbon pool still need to be elucidated (Blankinship et al., 2018).

The SOC storage mechanisms could be identified *via* two main methods for a long time due to physical protection by aggregates from microbial organisms and chemical protection by mineral materials through organo-mineral complexes (Han et al., 2016). Soil aggregates could successfully use barriers to keep SOC from being accessible to microorganisms (Six et al., 2004). Soil aggregates can be divided into macroaggregates (>250 μm) and microaggregates (53–250 μm) by their sizes, formation mechanism and properties (Elliott, 1986). Mineral-associated organic carbon (MAOC) is formed by mineral components and SOC molecules *via* ligand exchange or van der Waals forces (Bai et al., 2018). Although previous studies have researched aggregates and MAOC, no obvious ground for their formation or composition has been found (Blankinship et al., 2018).

SOC can be divided into microbial-derived and plant-derived SOC by its origin (Angst et al., 2021). In general, plant residue is considered the main source of SOC formation, and soil microbes act as decomposers but do not contribute (Kogel-Knabner, 2017). The plant-derived SOC has been decomposed by soil microbial organisms from the plant residues. Soil microbial organisms can also utilize plant residues by biosynthesis for growth. Ultimately, the carbon contained in the microbial necromass enters the soil carbon pool by the entombing effect (Liang et al., 2017; Liang, 2020). The current consensus is that microbial-derived materials play a vital role in stabilizing the SOC pool (Kallenbach et al., 2016; Cotrufo et al., 2019). Although microbial biomass turnover is fast in soils, the proportion of microbial biomass carbon constitutes only a small fraction of SOC. Microbial necromass carbon is seen as a fraction of the stable carbon pool (Liang et al., 2017). Amino sugars are typical biomarkers that contain substances in the cell walls of microbial necromass. Amino sugar measurements are important detection methods for researching the presence of soil microbial necromass (Amelung et al., 2008). Amino sugars are almost negligible in plant residues. Amino sugars in soil are very complicated; the most important categories of amino sugars comprise glucosamine (GluN), muramic acid (MurA), mannosamine (ManN) and galactosamine (GalN) (Zhang and Amelung, 1996). Specifically, MurA is a typical marker of bacterial necromass, as it only occurs in bacterial cell walls; chitin in fungal cell walls is the major component of GluN; and GluN is also found in bacterial necromass (Wang et al., 2021). The origin of GalN is still unclear, so GalN is frequently considered nonspecific. ManN also originates from bacteria and fungi, but distinguishing its origin from bacteria or fungi is still difficult (Liang et al., 2007). In recent years, researchers have recognized the importance of the contributions of microbial necromass to the stable SOC pool, which might be more than 50% in croplands (Wang et al., 2021).

SOC contents are indicators of soil productivity and sustainability because SOC acts as the carbon source for microbes and is critical for retaining soil fertility and productivity (Lal, 2016). Maintaining SOC contents at a relatively high level is essential for maintaining soil fertility and productivity. Soil microbial necromass carbon could contribute to half of the SOC contents in the global cropland (Wang et al., 2021). Therefore, microbial necromass carbon also plays an important role in remaining soil fertility and productivity. Consecutive maize straw mulching has been shown to increase both maize yield and soil microbial necromass carbon contents in a previous study (Liu et al., 2020). To date, no obvious evidence has shown the linkage between microbial necromass carbon and maize growth. The accumulation of microbial necromass carbon could increase the soil stable carbon pool as a result of the entombing effect. This would be beneficial for SOC storage, and higher SOC would be better for soil quality and productivity. Northeast China is situated at one of the golden maize belts and is a main grain-producing area. Intensive cultivation and a growing demand for food due to increased population have led to soil degradation and the decline in SOC (Lal, 2009). Suitable practices are needed to improve SOC and maintain soil fertility. Straw returning is an effective way to enhance soil fertility and SOC contents (Zhao et al., 2018; Tian et al., 2020). However, straw return would lead to more greenhouse gas emissions and adverse carbon neutralization. Therefore, turning straw resources into biochar is an effective method for carbon storage. Biochar is a carbon-rich solid product produced by biomass *via* pyrolysis and oxygen-limited conditions (Chen et al., 2019). Biochar usually contains a large amount of carbon, and the carbon in biochar is mainly aromatic carbon (Chen et al., 2019). This extremely stable carbon has stayed in soils from millennial to centennial timescales. At the very beginning of the study, biochar was used as a soil amendment to enhance soil organic carbon sequestration due to its large carbon sequestration potential (Bolan et al., 2021). However, biochar has multifunctional values beyond carbon storage in actual production, such as porous materials for mitigating greenhouse gas emissions, catalysts in industry, nanomaterials in industry, feed supplements in animals to improve animal health, and even immobilizing agents in organic contaminants and heavy metals in soil and water (Kumar et al., 2020; Bolan et al., 2021; Lin et al., 2022). Several studies have researched the effect of biochar on microbial necromass. A 34-month incubation study investigated the metabolic traits of microbial communities in aged biochar, and the results indicated that biochar has the potential to protect SOC by mediating bacterial metabolic capacities (Sun et al., 2016). Soil microbial activity can also be enhanced by the application rate of biochar doses, and the stability of microbial necromass is also well-maintained by biochar amendments (Zhang et al., 2021a,b). Previous studies have shown that biochar can stimulate soil microbial activity and improve MBC (Yang et al., 2017b; Fang et al., 2018; Dai et al., 2021). However, little attention has been given to the effect of biochar on living microbial organisms and dead necromass and the contribution of and relationship among MBC, microbial necromass carbon and SOC. The objectives of this study were to investigate (1) the effects of maize straw and biochar on soil MBC, POC, MAOC, DOC and microbial necromass carbon, (2) the contribution of MBC and microbial necromass carbon to the SOC pool and (3) the relationships among soil microbial community structure, microbial necromass carbon and other SOC fractions under maize straw and biochar application for nine consecutive years.

## Materials and methods

### Field experimental site and experimental design

The field experiment was conducted at the Shenyang Agricultural University field experiment station (41°49′N, 123°33′E) starting in May 2013. This station is situated in Northeast China, one of the three gold maize belts of the word. The climate is a warm continental monsoon climate. The frost-free period is ~ 150 days. The entire growth season is ~130–150 days. The annual average precipitation is approximately 705 mm, and the mean temperature is approximately 7.9°C. The soil type at this site is classified as Haplic Luvisols by WRB classification. The basic soil properties at the beginning of the experiment are shown in Yang et al. (2017a). The 9-year field experiment was conducted from May 2013 to October 2018. The field experiments included three treatments: CK (mineral NPK fertilizer applied only), BC (biochar applied annually at a rate of 2.625 t ha^−1^ together with mineral NPK fertilizer), and SR (maize stover returned at a rate of 7.5 t ha^−1^ together with mineral NPK fertilizer). The biochar application rate was according to the 35% inversion rate of maize stover biomass of 7.5 t ha^−1^ charred during pyrolysis. The mineral NPK fertilizer was applied at rates of urea (120 kg N ha^−1^), calcium superphosphate (60 kg P_2_O_5_ ha^−1^) and potassium sulfate (60 kg K_2_O ha^−1^). All fertilizers were applied once before sowing. The cropping pattern was continuous maize cropping. The area of each plot was 3.6 × 10 m^2^. Three replicates of each treatment were arranged in a randomized block design.

### Maize stover and biochar

The biochar applied in the study was produced by Jinhefu Agriculture Development Company, Liaoning, China. The pyrolysis conditions were ~450°C, and the pyrolysis duration lasted 90 min. Maize stover was collected from the field and then broken down into pieces of 5–7 cm. Both biochar and maize stover were applied by hand on the soil surface. Subsequently, a rotary cultivator was used to uniformly mix the amendments with the soil. The initial properties of biochar and maize stover were detailed in Yang et al. (2017a).

### Sampling and analysis

Topsoil (0–20 cm) was collected in early October 2021 after nine growing seasons. Undisturbed soil samples were collected in each treatment for soil aggregate separation. Undisturbed soil samples were collected by the profile method (dig a profile, cut the undisturbed soil to a vertical depth of 20 cm, and then hold the samples in aluminum boxes) and from five randomly selected locations in all plots. Then, all samples from the same plot were mixed together and transported to the laboratory. Bumping was avoided to protect the undisturbed soil samples during transportation. During the air-drying process, the undisturbed soil samples were sieved through an 8 mm mesh. Then, the samples were stored for aggregate analysis. The wet-sieving method was used to measure water stable aggregate fractions (Elliott, 1986). Different aggregate fractions were separated by a series of sieves (2,000 μm, 250 μm and 53 μm). The four aggregate fractions were (1) >2,000 μm (large macroaggregates), (2) 250–2,000 μm (small macroaggregates), (3) 53–250 μm (microaggregate), and (4) < 53 μm (silt+clay fraction). The detailed procedure is shown in Sun et al. (2021).

Bulk soil samples were also collected from each plot at the same time. To achieve representative samples, five random sampling points were chosen in each plot. Topsoil (0–20 cm) was abundantly mixed together adequately and then placed in sealable plastic bags. These bulk samples are transported to the laboratory and divided into two parts. One part of the samples was stored fresh to analyze soil MBC and PLFAs. The other samples were air-dried and stored for the detection of amino sugars and other chemical properties.

### Determination of amino sugars

The amino sugar contents in soils were detected by the method described by Zhang and Amelung (1996). First, the air-dried soil samples were sieved through a 0.25 mm mesh. The sieved samples containing 0.3 mg N were hydrolyzed under N_2_ conditions with 6 M HCl (10 min) at 105°C for 8 h. The hydrolysate was filtered and dried with an evaporator. The samples were dissolved in deionized water, and the pH was adjusted to 6.6–6.8 by KOH (1 M) and HCl (0.01 M) solutions. Next, the supernatant was collected for freeze-drying, and the precipitate was removed by centrifugation (10,000 g, 10 min). Using methanol to wash the residues to recover the amino sugars, these amino sugars were transformed into aldononitrile derivatives that were extracted by 1.5 ml dichloromethane solution. The amino sugar derivatives were dissolved in 300 μL hexane and ethyl acetate solvent (v:v = 1:1) for final analysis until the removal of dichloromethane by N_2_. These amino sugar derivatives were separated on a Thermo ICS5000 ion chromatograph (ICS5000, Thermo Fisher Scientific, USA) equipped with a Dionex™ CarboPac™ PA20 column (150^*^3.0 mm, 10 μm). Soil total amino sugars were calculated by the sum of the MurA, GluN, ManN and Galn contents. MurA and GluN were used to calculate bacterial residue carbon and fungal residue carbon, respectively.

### Analysis of phospholipid fatty acids

The soil PLFA method was used to analyze the composition of the soil microbial community (Frostegard and Baath, 1996). In brief, fresh soil samples were freeze-dried by a vacuum freeze dryer (Labconco^*^ FreeZone). Freeze-dried soil (4 g) was extracted twice by a single-phase chloroform-methanol-citrate buffer (v:v:v = 1:2:0.8). All the supernatant was collected and mixed together as one sample. Then, chloroform and citric acid buffer were added, and the chloroform layer was separated after incubation overnight in the dark and dried with N_2_ at 30°C. Phospholipids were separated into neutral lipids, glycolipids and phospholipids by standard solid phase extraction (SPE) tubes (6 mL, 500 mg, Supelco Inc., Pennsylvania, USA). Then, the phospholipids were methylated by 1:1 methanol-toluene and 0.2 M KOH solution to transform into their respective fatty acid methyl esters. Methyl non-adecanoate fatty acid (19:0) was set up as an internal standard to quantify the concentrations of phospholipids before quantitative analysis of phospholipid fatty acids. The fatty acid methyl esters were identified by gas chromatography (Agilent 7890A, USA) equipped with MIDI peak identification software (Version 4.5; MIDI Inc., USA). The microbial community composition was classified according to phospholipid fatty acid markers, phospholipid fatty acids 16:1ω7c, 17:0 cyclo ω7c, 18:1ω7c, 19:0 cyclo ω7c, 15:0 iso, 15:0 anteiso, 16:0 iso, 17:1 iso w9c, 17:0 anteiso were used as biomarkers for bacteria; 18:2ω6c, 18:1ω9c, 16:1ω5c for fungi; and the sum of PLFA content was used to represent the total microbial PLFAs (Klamer, 2004; Bach et al., 2010; Landesman and Dighton, 2010; Xu et al., 2022).

### Determination of soil chemical properties and maize yield

One part of the fresh soil samples was used to determine the soil microbial biomass carbon. The determination of soil MBC was determined by the chloroform fumigation method (Vance et al., 1987). Briefly, fresh soil samples (equivalent to 10 g of oven-dried soil) were weighed in glass beakers. Then, the samples were fumigated and non-fumigated for 24 h at 25°C in the dark. After the fumigated and non-fumigated processes, all samples were extracted by K_2_SO_4_ solutions (0.5 M) immediately. After shaking and centrifuging all the extracted samples, the supernatant was filtered through a 0.22 μm filter and detected by a TOC analyzer (Multi C/N 3100, Analytik Jena, Germany). Soil organic carbon and aggregate-associated organic carbon were detected by an Elementar Vario max Analyzer (Elementer Macro Cube, Germany) after sieving through a 0.15 mm mesh. The soil organic carbon fraction was isolated by density fractionation as described by Fang et al. (2018). Soil DOC was extracted by deionized water as described by Dong et al. (2019). Briefly, 10 g air-dried samples were weighed in flasks, and 50 ml deionized water was added to all flasks. All flasks containing soil samples were placed on a shaker and shaken at 230 rpm for 30 min. Then, all flasks were centrifuged at 4,000 × g for 40 min. The supernatant was filtered through a 0.45 μm filter and analyzed. All filtered supernatants were detected by a TOC analyzer (Multi C/N 3100, Analytik Jena, Germany). POC and MAOC were separated by a 1.8 g cm^−3^ sodium iodide solution. Briefly, 10 g of air-dried soil samples (sieved through 1 mm mesh) were weighed in one plastic centrifuge tube, and then 50 mL of 1.8 g cm^−3^ sodium iodide solution was added to the centrifuge tube. After shaking on a reciprocating shaker and centrifuging in a low-speed centrifuge, all the supernatant with floating particles was collected and filtered by a glass-fiber filter. The NaI solution was collected for reuse. This process was repeated twice as shown before. The floating samples that were filtered were washed with deionized water three times, and this fraction was POC. The residues in the centrifuge tube were also washed with deionized water three times to remove the residue NaI. The residue fraction in the centrifuge tube was MAOC. All POC and MAOC samples were oven-dried at 60°C until constant weight. All samples were weighed and stored for analysis. The POC and MAOC were also measured by an Elementar Vario max Analyzer (Elementer Macro Cube, Germany). At harvest, all ears of maize plants in the middle two rows in each plot were collected to measure the maize yield.

### Calculations and statistical analysis

Soil aggregate stability is traditionally expressed by the mean weight diameter (MWD), geometric mean diameter (GMD) and macroaggregates (R_>250μ*m*_) (Mazurak, 1950; van Bavel, 1950). The calculation equation is displayed as follows:


(1) $$\begin{array}{lll} MWD & = & \frac{\sum_{i=1}^{n}xiWi}{\sum_{i=1}^{n}Wi} \end{array}$$

(2) $$\begin{array}{lll} GMD & = & EXP\left[\frac{\sum_{i=1}^{n}milnxi}{\sum_{i=1}^{n}mi}\right] \end{array}$$

where *x*i is the average diameter of every aggregate fraction, *Wi* is the weight percentage of every aggregate fraction, and mi is the weight of different aggregate fractions.

Soil MBC was calculated by the following equation:


(3) $$\begin{array}{l} MBC\;\left(mg\;kg^{-1}\right)=\\ \;\frac{\text{Extracted }C_{fumigated\;soil}-{Extracted\;C}_{non-fumigated\;soil}}{K} \end{array}$$

where K is a correction factor of 0.45 (Vance et al., 1987).


(4) $$\begin{array}{l} fungal\;necromass\;carbon\;\left({mg\;g}^{-1}\right)=\\ \left(\;GluN/179.17-2\times mmol\;MurA/251.23\right)\times 179.17\times 9 \end{array}$$

(5) $$\begin{array}{l} bacteria\;necromass\;carbon\;\left({mg\;g}^{-1}\right)=MurA\left(mg\;g^{-1}\right)\times 4\text{5} \end{array}$$

where 179.2 is the molecular weight of GluN, 251.23 is the molecular weight of MurA, 9 is the conversion coefficient of fungal GluN to fungal necromass carbon, and 45 is the conversion coefficient from MurA to bacterial necromass carbon in equation 5 (Appuhn et al., 2006).

All data were processed by Office Excel 2016 and are expressed as the mean ± standard deviation. One-way analysis of variance (ANOVA) was used to test the differences among treatments. Multiple comparisons were performed by the least significant difference (LSD) method using IBM SPSS 22.0 software (New York, USA). All figures were generated by Origin 2022 software (Origin Lab Inc., Northampton, USA).

## Results

### Effects of maize straw and straw biochar on SOC contents

**Figure 1:**
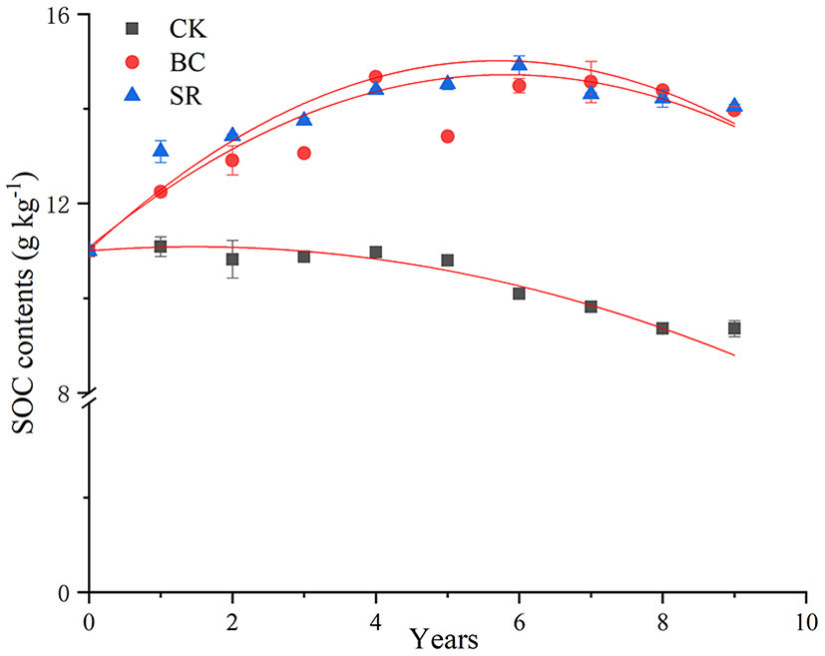
Dynamics of SOC contents during straw and biochar application for 9 consecutive years. The data of the first 3 years was cited from Yang et al. (2017a). Bars represent standard deviations (*n* = 3).

As shown in Figure 1, from 2013 to 2018, the SOC contents in the topsoil layer (0–20 cm) showed different dynamics. BC and SR enhanced the SOC content with annually applied organic materials, but the SOC contents decreased in the CK treatment compared to the initial level during the field experiment. The SOC level dynamics exhibited two separate stages in BC and SR, with a rapid accumulation stage (2013–2016) and a slow fluctuation stage (2017–2021).

### Effects of maize straw and straw biochar on SOC fractions

In this study, biochar and straw amendments caused significant changes in different SOC fractions after 9-year field experiments (Table 1). BC and SR enhanced the DOC contents by 116.59 and 162.70%, respectively. The proportions of DOC accounting for SOC also increased in the BC and SR groups compared to the CK group (Table 2), and the ratio of DOC/SOC followed the trend SR = BC>CK, which indicated that the ratio of DOC/SOC in the BC and SR groups was significantly higher than that in the CK group.

**Table 1:** Effect of straw and straw biochar on SOC fractions.

**Treatments**	**DOC (mg kg^−1^)**	**MBC (mg kg^−1^)**	**POC (g kg^−1^)**	**MAOC (g kg^−1^)**
CK	34.16 ± 2.99b	114.86 ± 14.71b	2.65 ± 0.01c	6.55 ± 0.16c
BC	73.99 ± 14.09a	170.62 ± 9.89a	5.36 ± 0.10a	8.42 ± 0.19b
SR	89.74 ± 4.96a	184.73 ± 13.39a	4.57 ± 0.10b	9.22 ± 0.10a

Different lowercase letters indicate significant differences (P < 0.05) among different treatments. Data are means ± standard deviations, n = 3. SOC, soil organic carbon; MBC, microbial biomass carbon; POC, particulate organic carbon; DOC, dissolved organic carbon; MAOC, mineral-associated organic carbon.

**Table 2:** The proportions of different SOC fractions accounting for SOC contents.

**Treatments**	**DOC/SOC**	**MBC/SOC**	**POC/SOC**	**MAOC/SOC**
	**%**			
CK	0.36 ± 0.03b	1.23 ± 0.14a	28.36 ± 0.51c	70.01 ± 0.51a
BC	0.53 ± 0.10a	1.22 ± 0.07a	38.40 ± 0.93a	60.28 ± 0.99b
SR	0.64 ± 0.03a	1.32 ± 0.10a	32.51 ± 0.20b	62.61 ± 0.29b

Different lowercase letters indicate significant differences (P < 0.05) among different treatments. Data are means ± standard deviations, n = 3.

Compared to that in the CK group, the MBC contents increased by 48.54% and 60.83% in the BC and SR treatments, respectively. The contents of MBC in the BC and SR groups were non-significantly different after the 9-year field experiment (*P* > 0.05). The ratio of MBC/SOC was non-significantly different among the three treatments (*P* > 0.05).

BC and SR significantly enhanced soil POC (Table 1). The soil POC contents increased by 102.09 and 72.19% in the BC and SR groups, respectively. The proportions of POC accounting for SOC followed the trend BC>SR>BC, which indicated that the ratio of POC/SOC was higher in the BC group than in the SR and CK groups.

The MAOC contents were significantly enhanced by the BC and SR treatments after the 9-year field study (Table 1). The MAOC contents followed the trend SR>BC>CK. The BC and SR treatments increased the MAOC contents by 28.49 and 40.70%, respectively. The MAOC/SOC ratio followed the trend of CK>SR=BC, which indicated that the MAOC proportion of SOC was higher in the CK group than in the BC and SR groups (Table 2).

### Effects of maize straw and straw biochar on soil amino sugars and microbial necromass carbon

After the 9-year field experiment, soil total amino sugars and different amino sugars showed different trends (Table 3). The GalN content was the highest in the SR group among the three treatments, followed by the BC treatment and the CK group. Compared to that in the CK group, the content of GalN increased by 20.29 and 41.63% in the BC and SR groups, respectively. The ManN content followed the trend SR>CK>BC. The ManN content was highest in the SR group, followed by the CK group and the BC group. The GluN content had the same trend as the GalN content, which followed the trend SR>BC>CK; the BC and SR treatments enhanced the GluN content by 24.54 and 54.91%, respectively. BC treatment had no effect on the MurA content after the 9-year field experiment, SR treatment significantly enhanced the MurA content in the current study, the MurA content was significantly higher in the SR group than in the BC and CK groups (*P* < 0.05), and no significant difference was observed in the MurA content between the CK and BC groups (*P* > 0.05). The soil total amino sugar content was calculated by the contents of the above amino sugars; the total amino sugar content was highest in the SR group, followed by the BC group and the CK group. Compared to that in the CK group, the total amino sugar content increased by 18.18 and 42.71% in the BC and SR groups, respectively.

**Table 3:** Effect of straw and straw biochar on soil amino sugars.

**Treatments**	**GalN (mg kg^−1^)**	**ManN (mg kg^−1^)**	**GluN (mg kg^−1^)**	**MurA (mg kg^−1^)**	**Total amino sugars (mg kg^−1^)**
CK	400.43 ± 0.51c	123.02 ± 0.50b	432.23 ± 0.12c	27.67 ± 0.50b	983.36 ± 1.04c
BC	481.68 ± 0.69b	114.88 ± 0.18c	538.31 ± 0.92b	27.24 ± 0.48b	1,162.11 ± 1.83b
SR	567.12 ± 0.31a	134.36 ± 0.70a	669.56 ± 0.33a	32.31 ± 1.20a	1,403.35 ± 0.71a

Different lowercase letters indicate significant differences (P < 0.05) among different treatments. Data are means ± standard deviations, n = 3. Galn, galactosamin; GluN, glucosamine; MurA, muramic acid; ManN, manosamine.

Bacterial-derived carbon and fungal-derived carbon are shown in Table 4. After the 9-year field experiment, no significant differences in bacterial-derived carbon content were observed between the BC and CK groups (*P* > 0.05), the bacterial-derived carbon content in the SR group was significantly higher than that in the BC and CK groups (P < 0.05), and the bacterial-derived carbon content in the SR group increased by 16.80 and 18.64% compared to that in the CK and BC groups, respectively. Both BC and SR treatment enhanced the fungal-derived carbon content compared to the CK group (*P* < 0.05). Compared to that in the CK group, the fungal-derived carbon content increased by 27.16% in the BC group and 58.74% in the SR group. Soil microbial necromass carbon was calculated by the sum of bacterial-derived carbon and fungal-derived carbon. The soil microbial necromass carbon followed the trend of SR>BC>CK. Compared to the CK treatment, the BC and SR treatments enhanced the microbial necromass carbon by 19.68 and 47.81%, respectively.

**Table 4:** Effect of straw and straw biochar on soil microbial necromass carbon contents.

**Treatments**	**Bacterial-derived carbon g kg^−1^**	**Fungal-derived carbon g kg^−1^**	**Microbial necromass carbon g kg^−1^**
CK	1.24 ± 0.10b	3.53 ± 0.02c	4.78 ± 0.15c
BC	1.23 ± 0.10b	4.50 ± 0.03b	5.72 ± 0.20b
SR	1.45 ± 0.01a	5.61 ± 0.10a	7.07 ± 0.12a

Different lowercase letters indicate significant differences (P < 0.05) among different treatments.

The ratio of microbial necromass carbon accounting for SOC and MAOC is shown in Table 5. We found that the ratio of microbial necromass carbon accounting for SOC and MAOC in the BC group was significantly lower than that in the CK and SR groups (*P* < 0.05). The difference in the ratio of microbial necromass carbon accounting for SOC and MAOC between the CK and SR groups was not significant (*P* > 0.05).

**Table 5:** The ratio of microbial necromass carbon accounting for SOC and MAOC.

**Treatments**	**Microbial necromass carbon/SOC %**	**Microbial necromass carbon/MAOC%**
CK	51.08 ± 0.92a	72.98 ± 1.84a
BC	40.96 ± 0.25b	67.97 ± 1.51b
SR	50.28 ± 0.38a	76.64 ± 0.89a

Different lowercase letters indicate significant differences (P < 0.05) among different treatments.

### Effects of maize straw and straw biochar on soil aggregates

**Figure 2:**
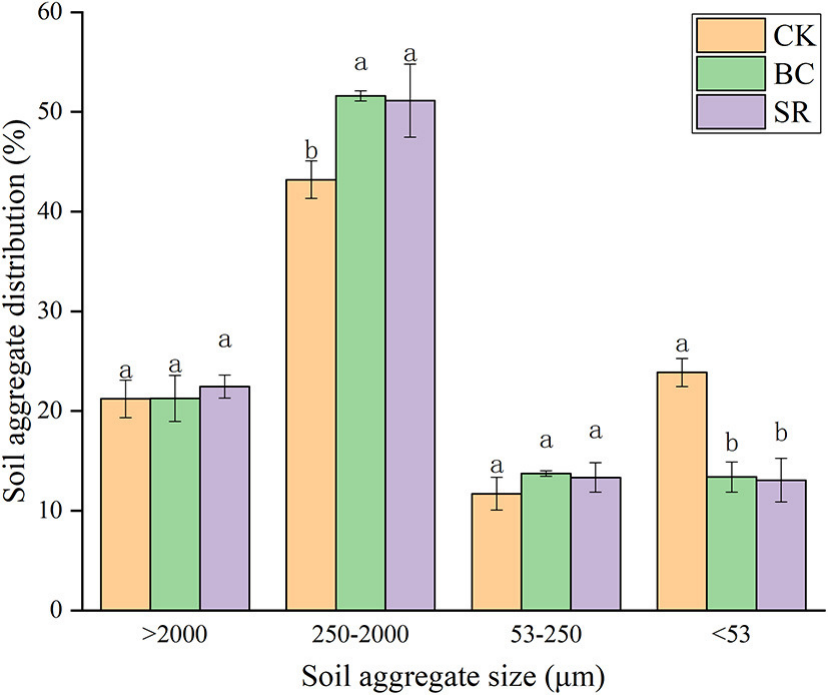
Effect of maize straw and straw-derived biochar on soil aggregate distribution in Year 2021. The error bars represent the standard deviations of the mean values (*n* = 3). Different lowercase letters indicate significant differences between the different treatments.

As shown in Figure 2, small macroaggregates (250–2,000 μm) dominated in brown earth, and the proportions of these small macroaggregates ranged from 43.20~51.61%. The microaggregate proportions were the lowest of all the aggregate fractions, ranging from 11.71~13.73%. No differences were observed in large macroaggregates and microaggregates among treatments (*P* < 0.05). Compared to that in the CK group, the proportion of small macroaggregates increased by 19.46 and 18.37% in the BC and SR groups, respectively. No differences were observed in the small macroaggregate fraction between the BC and SR groups (*P* < 0.05). Both the BC and SR treatments decreased the silt+clay fraction. Compared to that in the CK group, the silt+clay fraction decreased by 43.90 and 45.27% in the BC and SR groups, respectively.

The aggregate MWD and GMD in the BC and SR groups were higher than those in the CK group (Table 6). However, the difference in MWD among treatments was not significant (*P* > 0.05), the difference in GMD in the BC and SR groups was significantly higher than that in the CK groups (*P* < 0.05), and the BC and SR treatments increased the GMD by 42.24 and 47.58%, respectively. The macroaggregate content was also enhanced significantly by amendments (*P* < 0.05). Compared to that in the CK group, the macroaggregate content increased by 13.13 and 14.24% in the BC and SR groups, respectively.

**Table 6:** Effect of maize straw and straw-derived biochar on soil aggregate stability in 2021.

**Treatments**	**MWD mm**	**GMD mm**	**Macroaggregates%**
CK	1.57 ± 0.10a	0.50 ± 0.05b	64.42 ± 3.04b
BC	1.67 ± 0.11a	0.71 ± 0.07a	72.89 ± 1.78a
SR	1.72 ± 0.03a	0.74 ± 0.06a	73.60 ± 2.79a

The error bars represent the standard deviations of the mean values (n = 3). Different lowercase letters indicate significant differences between the different treatments. MWD, mean weight diameter; GMD, geometric mean diameter.

### Effects of maize straw and straw biochar on soil phospholipid fatty acids

**Figure 3:**
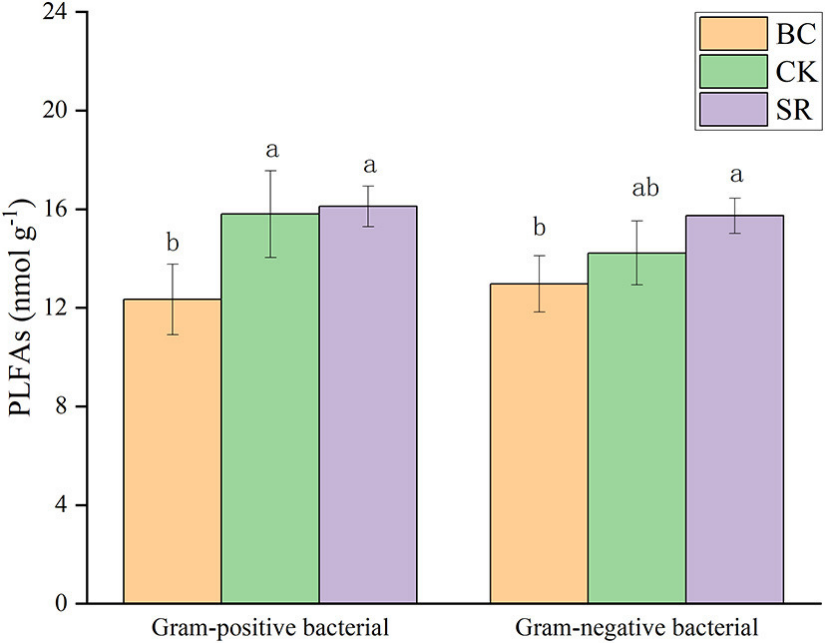
Effect of maize straw and straw-derived biochar on gram-positive and gram-negative microbial PLFAs. The error bars represent the standard deviations of the mean values (*n* = 3). Different lowercase letters indicate significant differences between the different treatments.

As shown in Figure 3, soil total microbial PLFAs were significantly increased by biochar and straw application (*P* < 0.05), but no difference was observed between the BC and SR groups (*P* > 0.05). Soil microbial PLFAs dominated in all treatments over fungal PLFAs and actinomycetes PLFAs (average of 29.26 vs. 7.34 and 9.01 nmol g^−1^). Both BC and SR enhanced soil bacterial PLFAs, but only SR significantly enhanced microbial PLFAs (*P* < 0.05). Both BC and SR enhanced the soil fungal PLFA content, which followed the trend of SR>BC>CK. The soil actinomycete PLFA content showed the same trend as the bacterial PLFA content.

As shown in Figure 3, gram-positive bacterial and gram-negative bacterial PLFAs were significantly affected by biochar and straw application (*P* < 0.05). The BC and SR treatments significantly enhanced the gram-positive bacterial PLFAs (*P* < 0.05), and only the SR treatment significantly enhanced the gram-negative PLFA content. No differences were observed between the BC and SR treatments for either gram-positive or gram-negative PLFA contents (*P* > 0.05).

The BC and SR treatments both significantly increased the fungal/bacterial PLFA ratio in the current study (*P* < 0.05) (Table 7). Compared to that in the CK group, the ratio of fungal/bacterial PLFAs was increased by 14.96 and 20.95% in the BC and SR treatments, respectively. No significant differences in the ratio of total amino sugar/total PLFA contents were found among treatments (*P* > 0.05) (Table 7).

**Table 7:** The ratios of total amino sugars to total phospholipid fatty acids (PLFAs) for different treatments.

**Treatments**	**Fungi/Bacterial PLFA**	**Total amino sugars/total PLFAs**
CK	0.22 ± 0.01b	25.49 ± 2.80a
BC	0.26 ± 0.01a	24.99 ± 2.66a
SR	0.27 ± 0.00a	27.82 ± 1.33a

The error bars represent the standard deviations of the mean values (n = 3). Different lowercase letters indicate significant differences between the different treatments.

### Effects of maize straw and straw biochar on maize yields

**Figure 4:**
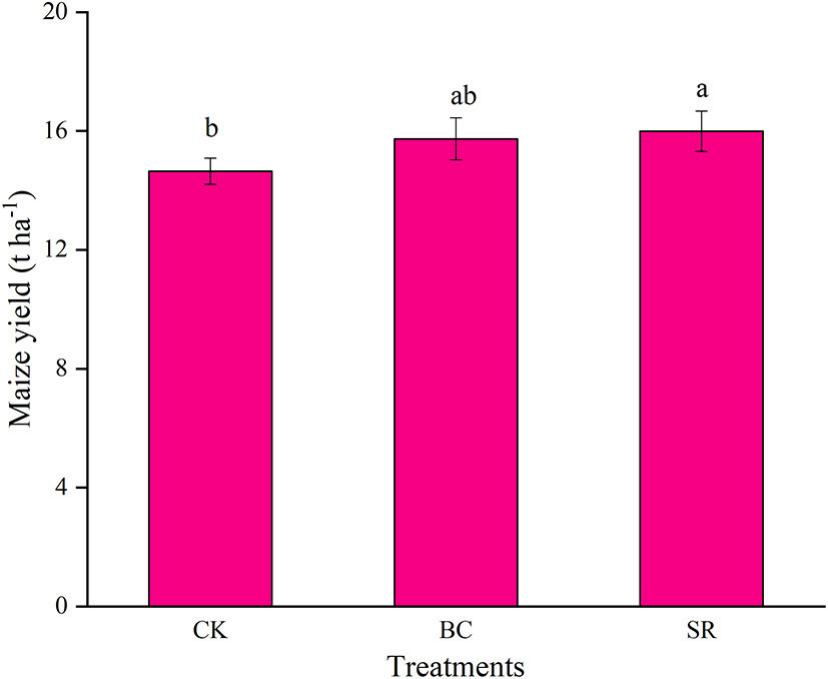
Effect of maize straw and straw-derived biochar on maize yield. The error bars represent the standard deviations of the mean values (*n* = 3). Different lowercase letters indicate significant differences between the different treatments.

As shown in Figure 4, maize yield were significantly increased by biochar and straw application (*P* < 0.05), but no difference was observed between the BC and SR groups (*P* > 0.05). Compared to CK, maize yield were increased by 7.44 and 9.16% in the BC and SR treatments, respectively. So biochar and straw could increase maize yield after nine-year field experiment.

### Relationships among soil properties

**Figure 5:**
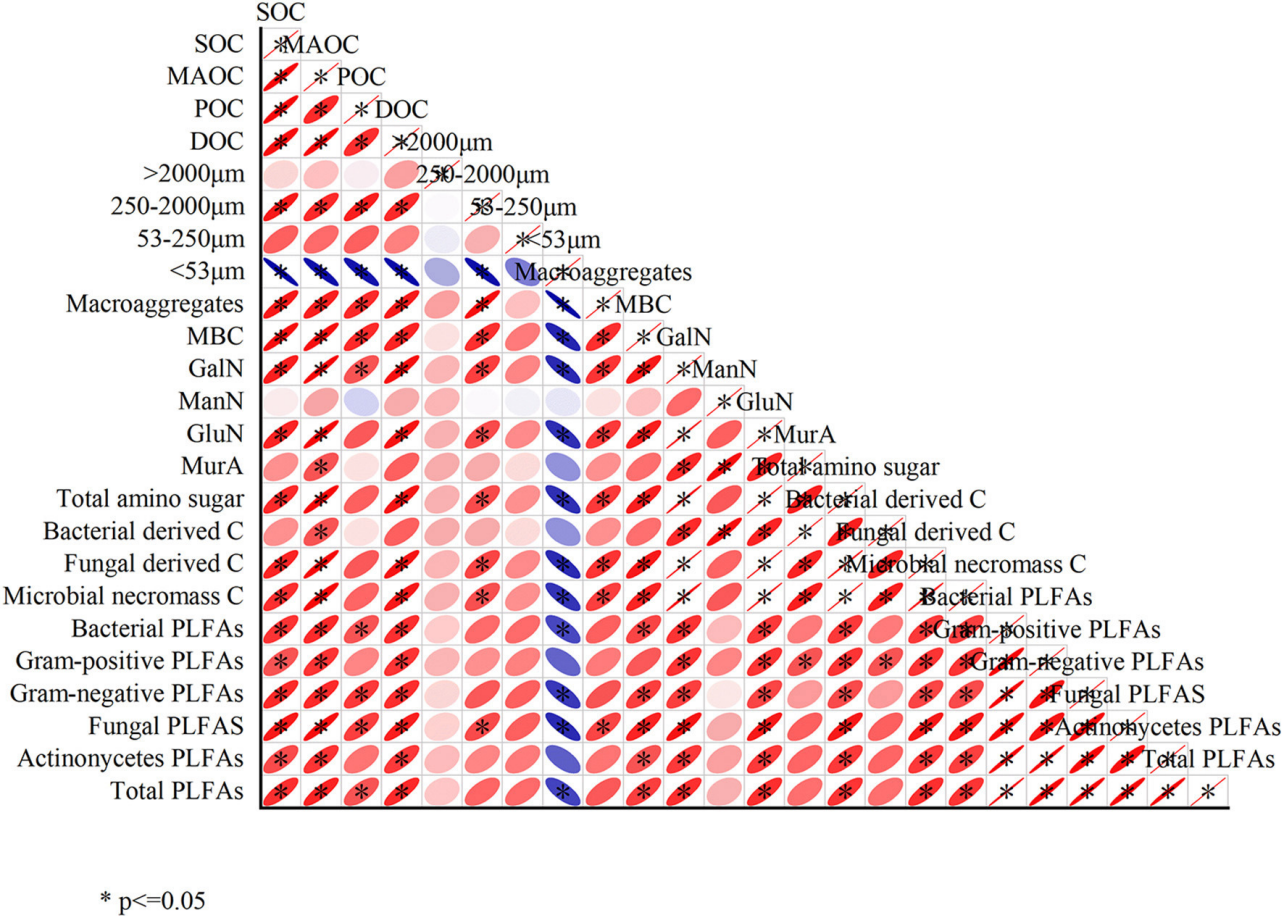
Relationships among different variables. *indicate significance at *p* < 0.05.

The correlations among different variables are shown in Figure 5. Almost all the variables showed a positive correlation except the silt+clay fractions (< 53 μm). SOC and MAOC were significantly positively correlated with GalN, GluN, total amino sugars, bacterial PLFAs, fungal PLFAs, actinomycetes PLFAs and total PLFAs (*P* < 0.05). The silt+clay fraction was significantly negatively correlated with SOC, MAOC, POC, DOC, macroaggregates, MBC, GalN, GluN, total amino sugars, fungal-derived C, microbial necromass carbon, bacterial PLFAs, fungal PLFAs, and total PLFAs.

**Figure 6:**
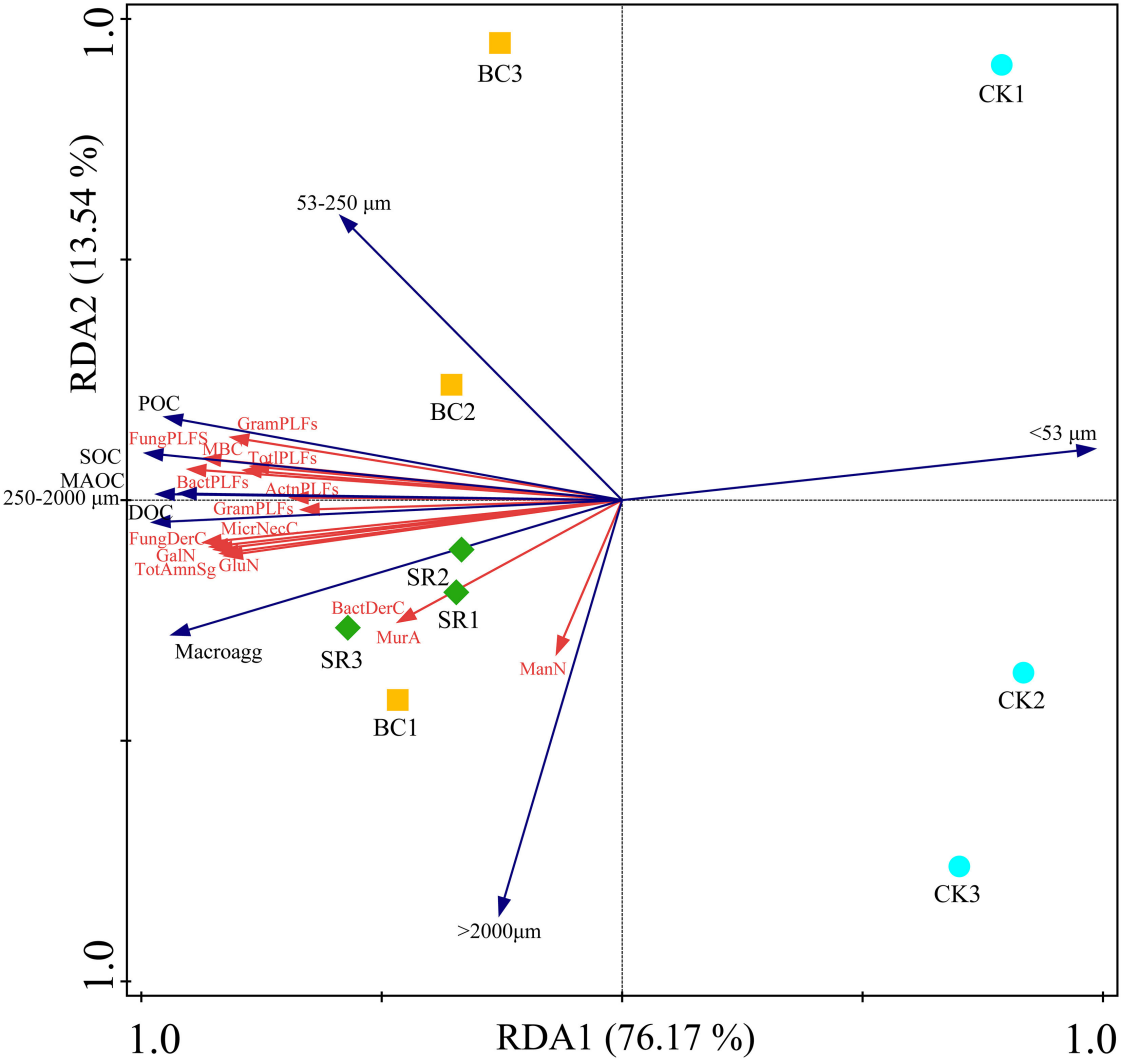
Redundancy analysis (RDA) showing the effect of different environmental variables on the microbial community.

The RDA results showed that the soil amino sugar content, microbial necromass carbon content and PLFA content were significantly related to the SOC and aggregate fractions (Figure 6). The environmental variables could explain 89.71% of the total variance.

## Discussion

### Long-term effects of maize straw and straw biochar on SOC dynamics and SOC fractions

Different straw management practices (such as straw return and biochar amendment) were useful ways to improve SOC contents in the plow layer. The SOC content decreased during the 9-year field experiment in the CK group, indicating that continuous planting would deplete SOC. SOC dynamics are vital for global food and nutritional security due to their nutrient supplementation (Lal, 2016). In the current study, both biochar and straw enhanced SOC contents during the 9-year field experiment. In the first 4 years (2013–2016), the SOC content increased rapidly in the BC and SR treatments, followed by a slow fluctuation period (Table 8). The SOC content is regulated by carbon input and the mineralization process (Cotrufo et al., 2015). The amount of carbon input was different in the BC and SR treatments. In theory, the amount of carbon input in the SR and BC groups was 3.22 t annually and 1.73 t annually, respectively. The SOC content was not significantly different between the BC and SR groups after the 9-year field study. This result indicated that biochar has more advantages in improving SOC in farmland because of the high stability of biochar carbon (Dong et al., 2016).

**Table 8:** The fitting equations in different treatments.

**Treatments**	**Fitting equation**	**R^2^**
CK	y = 11.0+0.1188x-0.0404x^2^	0.98
BC	y = 11.0+1.4036x-0.1229x^2^	0.96
SR	y = 11.0+1.2557x-0.1080x^2^	0.98

In a recent framework, the SOC pool was divided into two carbon pools (POC and MAOC) by physical properties, which could be better for SOC accrual and persistence (Castellano et al., 2015; Cotrufo et al., 2015). MAOC refers to the organic molecules that combine with minerals or aggregate within highly stable fine microaggregates (Leuthold et al., 2022). The possible mechanisms of the formation of MAOC include hydrogen bonding, cation bridging, anion exchange, ligand exchange, coulombic attraction, and van der Waals forces (Bai et al., 2018). In the current study, both biochar and straw significantly enhanced the MAOC content, and the MAOC content in the SR group was significantly higher than that in the BC group (Table 1). The results indicated that biochar will inevitably participate in the biogeochemical process in the soil. In previous studies, biochar particles could be sufficiently associated with minerals based on their superficial functional groups (Chia et al., 2012; Burgeon et al., 2021). Straw decomposed by soil microorganisms can release polysaccharides and organic acids (Jastrow, 1996). These polysaccharides and organic acids play a positive role in the formation of MAOC (Choudhury et al., 2014). Our results also indicated that straw return plays a positive role in the formation of MAOC and soil aggregate stability. In general, soil POC usually refers to the primary SOC fraction composed of structural materials derived from plants or microorganisms that have undergone decomposition and fragmentation but little to no depolymerization (Leuthold et al., 2022). However, biochar as a soil amendment could also add biochar particles to soil conditions. Biochar particles (pyrolysis organic carbon) can be transformed from plants by thermal or combustion processes, and these biochar particles contain highly condensed aromatic rings (Lehmann and Joseph, 2015). Strictly speaking, the POC fraction in the biochar treatment was not the same as the traditional POC fraction. However, by using the physical separation method, POC and MAOC could be separated and accurately studied as different fractions according to their properties. We could sufficiently study the distribution of different SOC fractions in distinct physical fractions. In this study, the BC treatment had the highest POC content and proportions accounting for SOC compared to the CK and SR treatments. This result could be explained by the highly condensed aromatic properties of biochar carbon. The proportion of MAOC accounting for SOC in the CK group was the highest of all three treatments, and the proportion of POC accounting for SOC in the CK group was the lowest of all three treatments. This result was similar to that of previous studies, in which it was found that most organic carbon was stored in the MAOC fraction, especially in soils with low organic carbon contents (Cotrufo et al., 2019). Our results suggest that biochar and straw amendments could improve both the POC and MAOC contents. In previous studies, the SOC stock showed no significant increase in response to long-term continuous organic amendment inputs; this phenomenon is defined by carbon saturation (Six et al., 2002; Feng et al., 2014). Carbon saturation is mainly reflected by MAOC (Cotrufo et al., 2019). Thus, biochar has more potential than straw in carbon sequestration as it increases both MAOC and POC contents.

Soil DOC has been shown to have an extremely fast turnover rate and easy degradability, so DOC is crucial to SOC turnover and CO_2_ emissions (Vila-Costa et al., 2020). Studies of the effect of biochar amendment on soil DOC have revealed distinct results. Dong et al. (2019) suggested that biochar applied once has little effect on DOC contents and composition after a 5-year field experiment. Yang et al. (2017b) reported that biochar application decreases the DOC content compared to the CK treatment. Biochar could also enhance the DOC content in both acidic and neutral soils (Smebye et al., 2016). Straw return has a positive effect on improving the DOC content (Ye and Horwath, 2017; Gmach et al., 2020). The effect of biochar on the DOC content still requires further research. In this study, biochar amendment played a similar role as straw in improving the DOC content, as both biochar and straw increased the DOC content compared to that in the CK group. No significant difference in DOC content was found between the BC and SR groups. Straw decomposed by soil microorganisms could release small organic molecules to improve the DOC content; biochar also contains dissolved organic carbon, which could enhance the DOC content. The soil DOC content was determined by the input and output of soil organic C under various biogeochemical processes, such as decomposition, sorption and leaching (Bolan et al., 2011). Biochar had different effects on the DOC content, and these distinct results were attributed to the differences in biochar type, soil type, climate and cultivation management. In this study, the increase in DOC content by biochar could be explained by biochar enhancing soil microorganism activity and the MBC content, so more DOC could be released from SOC by soil microbial decomposition.

### Effect of maize straw and straw biochar on soil aggregates

Soil aggregates are the basic units in soil and impact many soil functions because they determine nutritional element contents and spatial distribution, the interactions between the solid and liquid interphase, and heat flow and capacity (Yudina and Kuzyakov, 2019). Biochar applied as a soil amendment has led to inconsistent results in previous studies. Different results were usually attributed to the different biochar feedstocks, distinct soil types, experimental durations and environments. Straw return usually plays a positive role in soil aggregation. Because straw resources are easily decomposed by soil microbes, additional binding agents and biological binding agents are released during the decomposition process (Dai et al., 2019; Lian et al., 2022). Our study also showed the same trend as that shown in previous studies. Biochar also played a positive role in the soil aggregation process. Biochar as a soil amendment still contains large amounts of non-pyrolyzed organic residue, which can stimulate soil microbial activity (Wang et al., 2017). Biochar can also absorb labile organic carbon as the substrate of soil microbial organisms (Liang et al., 2010). Soil MBC is a kind of biological binding agent associated with the soil aggregation process (Guo et al., 2018). Biochar and straw both increased the MBC content in the current study. Increasing the MBC content *via* biochar application might be a way to increase the soil macroaggregate content and aggregate stability.

### Effect of maize stover and straw biochar on soil necromass carbon and PLFAs

In this study, organic amendments increased amino sugars after consecutive crop seasons, except for the ManN content (Table 3). Both biochar and straw could provide substrates for soil microbes and improve microbial activity Yang et al. (2017b). The organic amendments increased the MBC content in this study. Both biochar and straw increased the GalN and GluN contents in the order of SR>BC>CK. The DBX and GluN contents had high proportions of the total amino sugar content, which indicated that fungi were more impacted than bacteria by the organic amendments. GalN was once thought to be closely related to bacterial-derived carbon (Joergensen et al., 2010); however, fungi could contribute more GalN to the total amino sugar content than bacteria under some conditions (Engelking et al., 2007). Further research is needed to quantify the origin of GalN in the future. GluN mainly exists in chitin, and the decomposition of chitin was much slower than that of MurA (Ding et al., 2013), so the fungi-derived carbon content was higher than the bacterial-derived C content. Biochar had no significant effect on the MurA content compared to the CK treatment, but straw amendment significantly increased the MurA content. This result could be explained by the difference in the metabolisms of microbial communities or decomposition rates of MurA in the BC and SR treatments. Microbial necromass carbon is an important component contributing to the stable SOC pool (Liang et al., 2017). In the current study, biochar and straw both enhanced fungi-derived carbon and total microbial necromass carbon. Biochar had no effect on the bacterial-derived carbon contents compared to CK, and straw still increased the bacterial-derived carbon compared to the CK and biochar amendments (Table 4). Biochar amendment decreased the proportion of microbial necromass carbon accounting in SOC and MAOC, but no differences were observed between the straw amendment and CK treatment (Table 5). The proportions of microbial necromass carbon accounting for the SOC pool varied from 40.96 to 51.08%, and these results showed that microbial necromass carbon accounted for almost 50% in the current study. Although biochar had the potential to increase the microbial necromass carbon concentration, biochar amendments promoted carbon sequestration by POC and microbial necromass carbon in this study.

**Figure 7:**
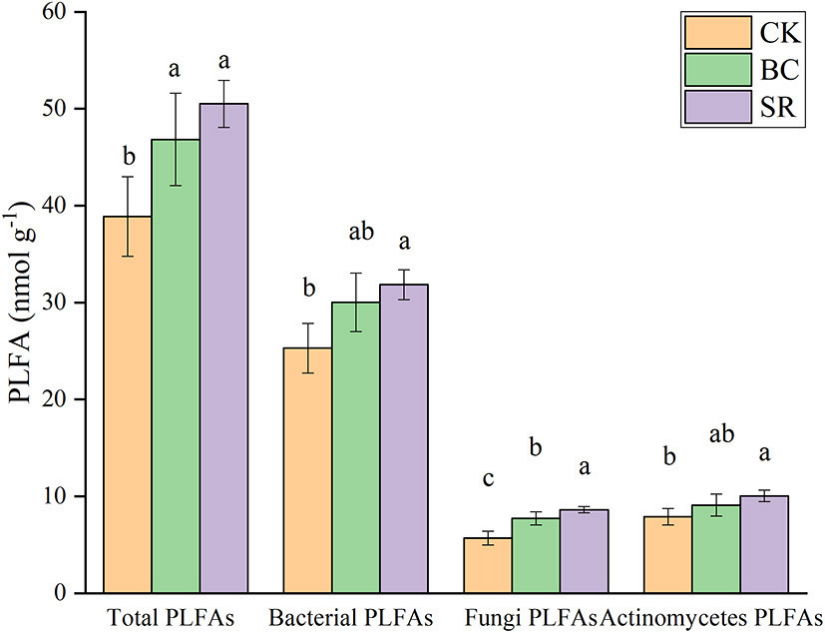
Effect of maize straw and straw-derived biochar on soil PLFA. The error bars represent the standard deviations of the mean values (*n* = 3). Different lowercase letters indicate significant differences between the different treatments. PLFA, phospholipid fatty acid.

Both biochar and straw as soil amendments increased the total PLFAs (Figure 7). These results indicated that biochar and straw could improve soil microbial activity and were significantly changed by organic amendments. Bacterial PLFAs were more abundant than other PLFAs (fungi, actinomycetes). This result was inconsistent with the microbial necromass carbon. Fungi-derived carbon was dominant in the soil microbial necromass carbon, but the bacterial PLFA content was dominant in the soil PLFAs. This result could be because the fungi-derived carbon was hard to decompose, but the bacterial-derived carbon was easy to decompose (Ding et al., 2013), as the cell walls of fungi were more recalcitrant than bacterial cell walls (Baldock and Skjemstad, 2000). Our results are similar to those of previous studies (Li et al., 2015).

## Conclusion

This study demonstrated that both biochar and straw had a positive effect on SOC dynamics and different SOC fractions after a 9-year field experiment. Biochar had the advantage of improving the POC content, but straw had the advantage of improving the MAOC. This result is due to the stable properties of biochar. Biochar and straw both increased the DOC and MBC contents, but no differences were observed between biochar and straw in DOC and MBC. This result indicates that biochar and straw could increase both the labile organic carbon fractions and microbial activity. Both biochar and straw increased the fungi-derived necromass carbon, total necromass carbon, fungi PLFAs and total microbial PLFAs. Biochar had no significant effect on the bacterial-derived necromass carbon and bacterial PLFAs. Maize yield increased by 7.44% and 9.16 after biochar and straw application for 9 years. Compared to straw, biochar could improve SOC mainly by fungal-derived necromass carbon and POC in the field.

## Data availability statement

The raw data supporting the conclusions of this article will be made available by the authors, without undue reservation.

## Author contributions

QS: conceptualization, data curation, formal analysis, investigation, methodology, resources, software, and writing-original draft. XY: conceptualization, data curation, formal analysis, and resources. ZB: formal analysis and investigation. JG: data curation and software. JM: supervision, writing–review and editing, project administration, and funding acquisition. XH, ZL, WC, and YL: supervision and writing-review and editing. All authors contributed to the article and approved the submitted version.

## Funding

This study was funded by the Earmarked Fund for Modern Agroindustry Technology Research System (No. CARS-01-51), the Innovative Talents Promotion Plan of Ministry of Science and Technology of the People's Republic of China (No. 2017RA2211), and the Project of Standardization Subsidy, Shenyang, China (2021-57).

## Conflict of interest

The authors declare that the research was conducted in the absence of any commercial or financial relationships that could be construed as a potential conflict of interest.

## Publisher's note

All claims expressed in this article are solely those of the authors and do not necessarily represent those of their affiliated organizations, or those of the publisher, the editors and the reviewers. Any product that may be evaluated in this article, or claim that may be made by its manufacturer, is not guaranteed or endorsed by the publisher.

## Abbreviations

SOC: soil organic carbon
MBC: microbial biomass carbon
POC: particulate organic carbon
DOC: dissolved organic carbon
MAOC: mineral-associated organic carbon
Galn: galactosamin
GluN: glucosamine
MurA: muramic acid
ManN: manosamine
PLFAs: phospholipid fatty acids
MWD: mean weight diameter
GMD: geometric mean diameter.
